# 3D pore pressure prediction in the offshore Nile Delta, prestack inversion based workflow for drilling optimization and risk mitigation

**DOI:** 10.1038/s41598-026-62603-2

**Published:** 2026-07-27

**Authors:** Mohamed Ahmed Abdelhay, Abdel Nasser Helal, Abdel Aleem Elessawy, Amir Lala

**Affiliations:** 1 Rashid petroleum Company (Rashpetco), Cairo, Egypt; 2 Ain Shams University, Cairo, Egypt; 3 Schlumberger Company, Cairo, Egypt

**Keywords:** Energy science and technology, Engineering, Solid Earth sciences

## Abstract

The offshore Nile delta basin, which is considered as one of the world’s high-pressure basins, is where the study develops the geopressured cube in 3D for the sapphire field. The key phase in the pressure prediction process is thought to be the interval velocity. In this study, we aim to use high-quality seismic data to estimate the shear and acoustic impedance volumes from the seismic pre-stack inversion technique. These cubes are then transformed into a high-resolution 3D interval velocity cube, which act as the initial and crucial input for the pressure estimation. Using four wells for model building and one for model evaluation, the workflow generated complete 3D cubes for overburden, effective stress, and formation pressure using the pre-stack inversion velocity. Optimizing the Eaton and Bowers equations parameter to be valid in the offshore Nile delta rather than the standard parameter from the Gulf of Mexico. it is considered One of the paper’s novelties. There is a high correlation between the pressure calculated from the logging while drilling (LWD) at the rig site and the formation pressure derived from the workflow. The technique shows a novel method for use in the well-planning and exploratory stages.

## Introduction

**Fig. 1:**
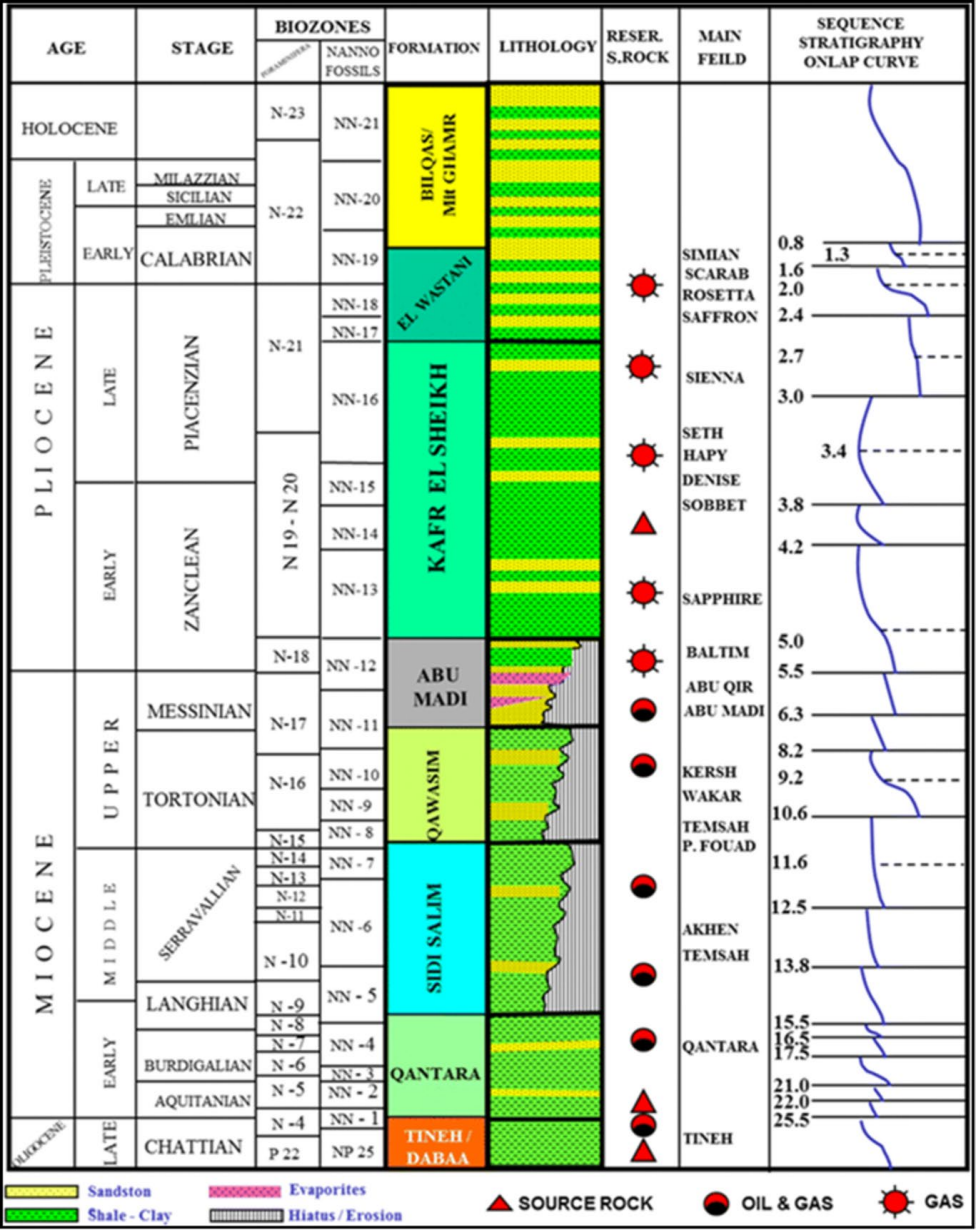
Nile Delta stratigraphic column and hydrocarbon system. Modified from Rio et al.^11^.

With 58 TCF of gas reserves, the Nile Delta region is considered as Egypt’s major gas province^1^. The Nile Delta’s geological sequence extends from the Holocene to Mesozoic ages (Fig. 1). Three main fault trends interact interactively to generate the structural configuration of this area. The Rosetta fault trend is NE-SW, the Temsah fault trend is NW-SE, and the E-W faults^2–4^. One of the producing areas within West Delta Deep Marine’s (WDDM) concession is our research area, Sapphire Field. Its stratigraphy is located in the lower Pliocene. It has several turbidite deposits within the shelf’s slope. The Nile Delta Offshore Anticline (NDOA) fault, which is located in the middle of WDDM, and the Rosetta fault, extended to the south-east corner of the concession, are the two main deep-seated faults that have an impact on the region’s structure.

Nile Delta basin. The offshore Nile Delta is recognized worldwide as an important example of over pressured basins. With a deep, clay-dominated Oligocene to Recent sedimentary layer, the Nile Delta is active geodynamic basins with a high rate of subsidence. Rapid sedimentation has led to the development of abnormally high formation pressures in both this section and the underlying pre-Tertiary section. Where the Messinian evaporite super seal is present, secondary mechanisms could possibly be locally placed. It is thought that variations in the volume of pore fluids or rock matrix resulting from either aqua-thermal expansion, hydrocarbon production, or thermal cracking of oil to gas in the lower Miocene upper Oligocene compartment are the cause of the sudden development of pore pressure in the southern portion of the Nile Delta. Compaction and heat forces are the primary causes of fluid flow in the Nile Delta^5^.

There are two main groups for the formation pressure: normal and abnormal. The normal pressure, also known as hydrostatic pressure, is the weight of the water column extended from a specific depth to the surface. The difference between the normal pressure and the formation pressure is known as the abnormal pressure^6^. When the formation pressure is higher than the normal pressure, overpressure results. Subnormal pressure, which happens when the formation pressure is lower than the normal pressure, is another kind of abnormal pressure. Abnormal pore pressure is caused by tectonics, fluid movement and buoyancy, fluid volume expansion, and compaction^7^. Abnormal formation pressure may originate from a variety of other factors, including tectonic activity, rapid deposition, reservoir structure, clay diagenesis, re-pressuring of shallow reservoirs, paleo pressure, salt domes, and density differences^8–10^.

The offshore Nile Delta, and specifically the Sapphire Field, has been the subject of extensive geological and geophysical research. Regarding the regional geological framework and stratigraphy, several studies have established the foundation for understanding the basin’s evolution; notably, sequence stratigraphic frameworks were advanced by Harwood et al., (1996) and Raslan (2002)^12,13^. Furthermore, the geological setting of the Sapphire Field has been detailed in key works by Taylor (2008)^14^, Abdelaziz et al.^15^, El-Kady (2017)^16^, and Salama)^17^.

In terms of seismic characterization, AVO and pre-stack inversion techniques have been extensively explored in the Nile Delta context (^18^; Radwan & Sharaf Eldin, 2014;^19,20^). Finally, pore pressure prediction methodologies have been significantly matured through numerous international and regional studies, including works by Olar^21^, Zi Wang (2014)^22^, Khalaf)^23^, El-Werr (2017)^24^, Bennett^25^, Tanko et al.^26^, Hutomo et al.^27^, Bahmaei and Hosseini^28^, Olayinka (2021)^29^, and more recently, Abdelhay (2025a, b)^30,31^. While this body of literature provides a robust baseline, this study builds upon these insights to address specific challenges in 3D pressure cube construction and regional parameter optimization.

## Methodology

**Pre-stack inversion** is a technique for integrating impedances from well logs with surface seismic amplitude data. To achieve a successful inversion, it is necessary to have strong well ties and high-quality seismic wavelets. Apply pre-stack inversion to generate P-impedance, S-impedance, Vp/Vs ratio, and density by using angle-limited stacks of the seismic data.

Zoeppritz’s 1919 equation is the first equation which links the reflection coefficient to the angle of incidence, VP, VS, and density. It considered as a basis of AVA (Amplitude Variations with Angle) by describe how amplitudes change with offset (angle) for elastic material, enabling a deduction of the physical properties of rocks across a seismic reflector. Appling Zoeppritz equations to actual seismic data is difficult due to the several variables to consider, using Zoeppritz equations to address subsurface challenges can be hard. Moreover, the seismic record is not composed of ideal reflected plane waves due to the complex structure of the earth. Researchers have developed simplified forms of the Zoeppritz equations to help with the application of AVO analysis. Aki and Richards^32^, Fatti^33^, Shuey^34^, Smith^35^, Verm^36^. They simplified the relation between reflection coefficient and angle of incidence to determine the main factors.

One of the simplified forms is Shuey equation. Shuey’s simplified formulation of the complex Zoeppritz equations displays how the reflection coefficient changes in relation to variations in impedance and Poisson’s ratio at different angles of incidence. The equation can be determined and calculated using conventional pre-stack data. Shuey^34^, modifies the Zoeppritz equations from Aki and Richards^32^ by changing the properties VS and ΔVS with σ and Δσ. The used equation is Shuey two-term (Eq. 1).

1 $$R(\theta)=R_{P0}+Gsin^2\theta$$

Where θ = angle of incidence, 0 = intercept and represents RC at normal incidence, G = gradient (slope of RC with offset or angle, (sin^2^ θ)).

While the Shuey equation is conventionally considered most accurate at angles below 30°, our analysis of the offshore Nile Delta dataset demonstrates that it remains effective at angles up to 45°.

**Pre-Drill pore pressure prediction**is A critical step in the drilling, production, and exploration stages is pre-drill pore pressure estimation. Terzaghi^37^, presents a simple formula for expressing relationship between overburden stress, effective stress, and pore pressure (equation-2).

2 $$S_{over}=S_{eff}+PP$$

The density log is used to calculate overburden stress. The density within the overburden was determined using two empirical formulae. In shallow depths between 500 and 700 m where hydrostatic pressure is present, density is determined using the Miller Equation (Eqs. 3 and 4).

3 $$\Phi=\Phi{a}+\Phi{b}\:EXP[-k\:(Dbml)(1/N)]$$

Where _a_ and _b_ is mudline porosity, k is fitting parameter, D is depth below mud line and N is fitting parameter.

4 $$\rho=\Phi\:\rho{w}+\rho{m}(1-\Phi)$$

Where is porosity, ρ_w_ is density for fluid (typically 1.03 g/cm3), ρ_m_ is density for fluid (typically 2.68 g/cm^3^).

The second equation, the Gardner Equation, uses the sonic or interval velocity for estimating the density of the other intervals^38^.

5 $$\rho=a\:V_{in}^b$$

Where V is the seismic interval velocity, a and b are lithology – dependent parameters (typically a = 0.23 & b = 0.25).

Pore pressure is predicted using the^39,40^ formulae based on interval velocity. The following assumed empirical relation between a vertical differential stress and velocity is the basis of Bowers’ method, which we applied to compute the normal velocity using the normal effective stress using the following equation:

6 $$V_n=V_o+A\sigma_n\:B$$

Where Vo is the compressional velocity at the mudline (usually 1524 m/s), A and B are the default constants from Gulf of Mexico (A = 10 and B = 0.57) and σn is the effective vertical stress at the normal condition.

Using Eaton’s approach, which is as follows, we can also calculate the vertical component of effective stress from seismic velocities:

7 $$\sigma_v=\sigma_n(V_{obs}/V_n)^{x}$$

Where σn and Vn are the vertical-differential stress and seismic velocity that would occur if the sediment was normally pressured, σv and Vobs are the vertical-differential stress and seismic velocity in situ, respectively, and x is an exponent which describes the sensitivity of velocity to differential stress. Following Eaton equation, x is constant.

Finally, the Terzaghi relation (Eq. 2) is used to calculate the Overburden Stress, Vertical Effective Stress, and Pore Pressure.

In this study, we using the interval velocity from the seismic pre-stack inversion approach as the initial input for the pore pressure prediction. The accuracy of these interval velocities directly affects the accuracy of this pore pressure calculation (Sayers (2002) and El-Werr (2017))^24,41^.

The novelty of this study lies in its integrated, multi-scale approach to pore pressure prediction in the complex geological environment of the offshore Nile Delta. Unlike traditional studies that rely on generalized parameters—often borrowed from the Gulf of Mexico—this research introduces a robust, calibrated workflow specifically optimized for the Sapphire field using the interval velocity extracted from the pre-stack inversion process which can be option when the seismic interval velocity absent^42–45^.

## Workflow & results

**Fig. 2:**
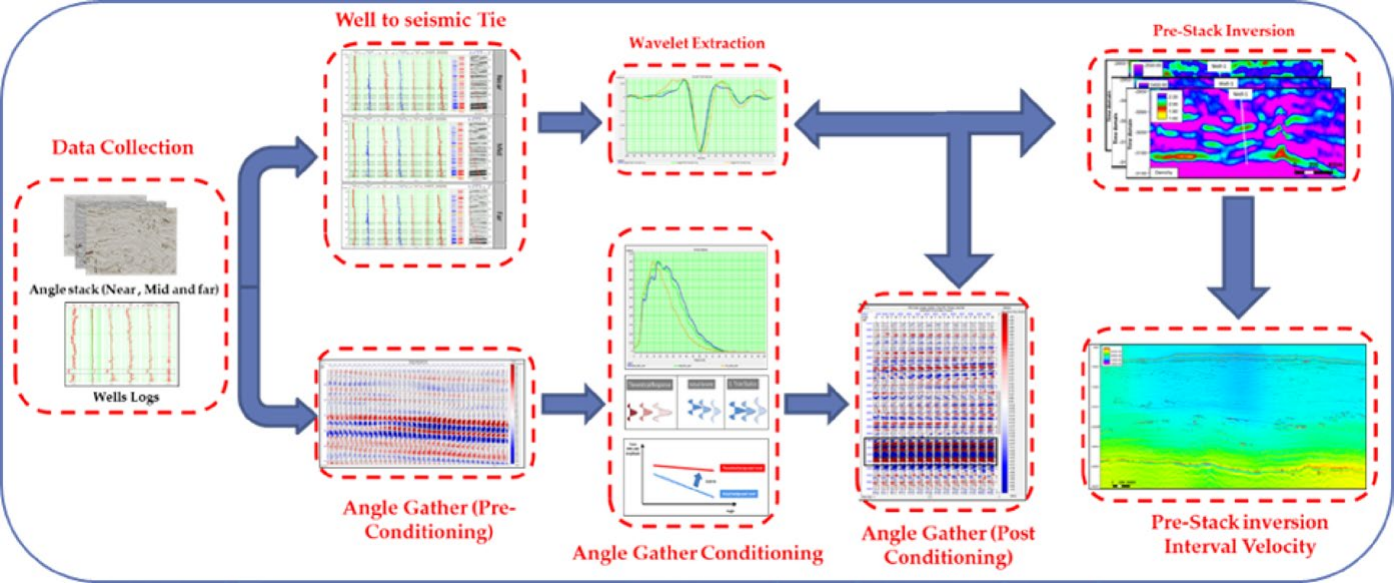
Seismic Pre-stack inversion workflow.

The work on this research is done on two phases. The first phase is the Seismic Pre-stack inversion work flow which started by seismic data conditioning and finish with the interval velocity estimation. The second phase which started by the interval velocity and finished by 3D pore pressure cube. Figure 2.

**Fig. 3:**
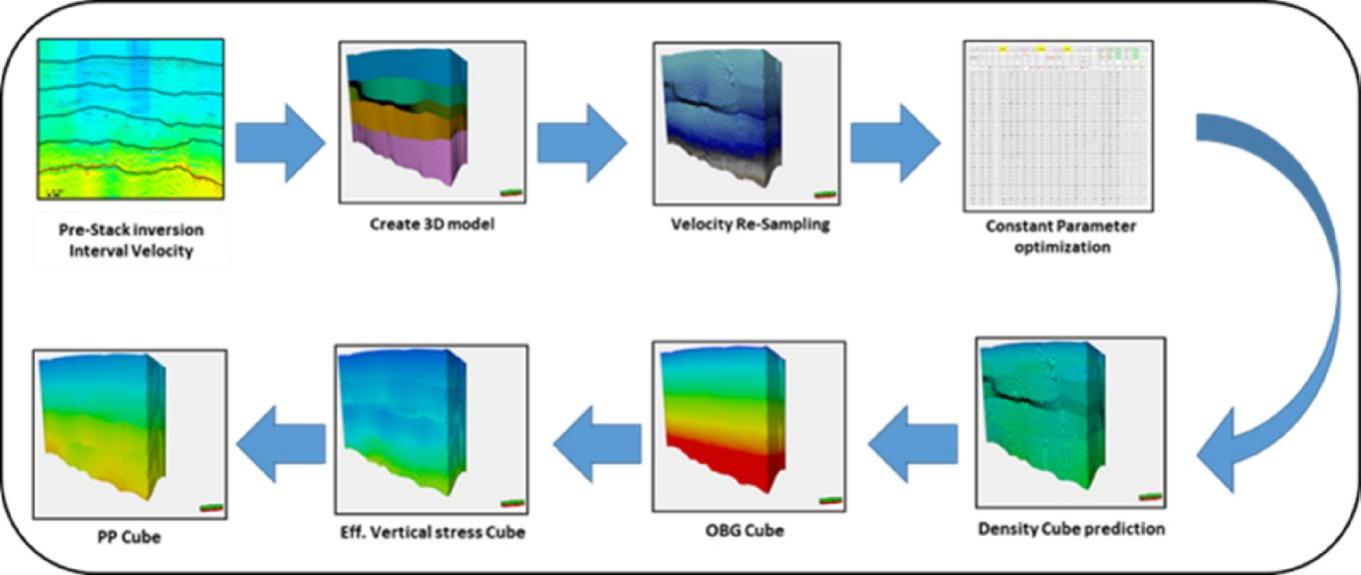
3D pore pressure modelling workflow.

The first step is data collection and preparation, which involve data gathering for five wells, four of which are used as model input and the fifth as a blinded well to evaluate the quality of the model. Wireline logs, seismic angle stack, and pressure logs, including overburden stress, effective vertical stress, and pore pressure logs, are examples of the data gathered. Cleaning the data and evaluating its validity are two elements of data preparation. The Blinded well was selected for well with only pore pressure data and no wire line logs.

**Fig. 4:**
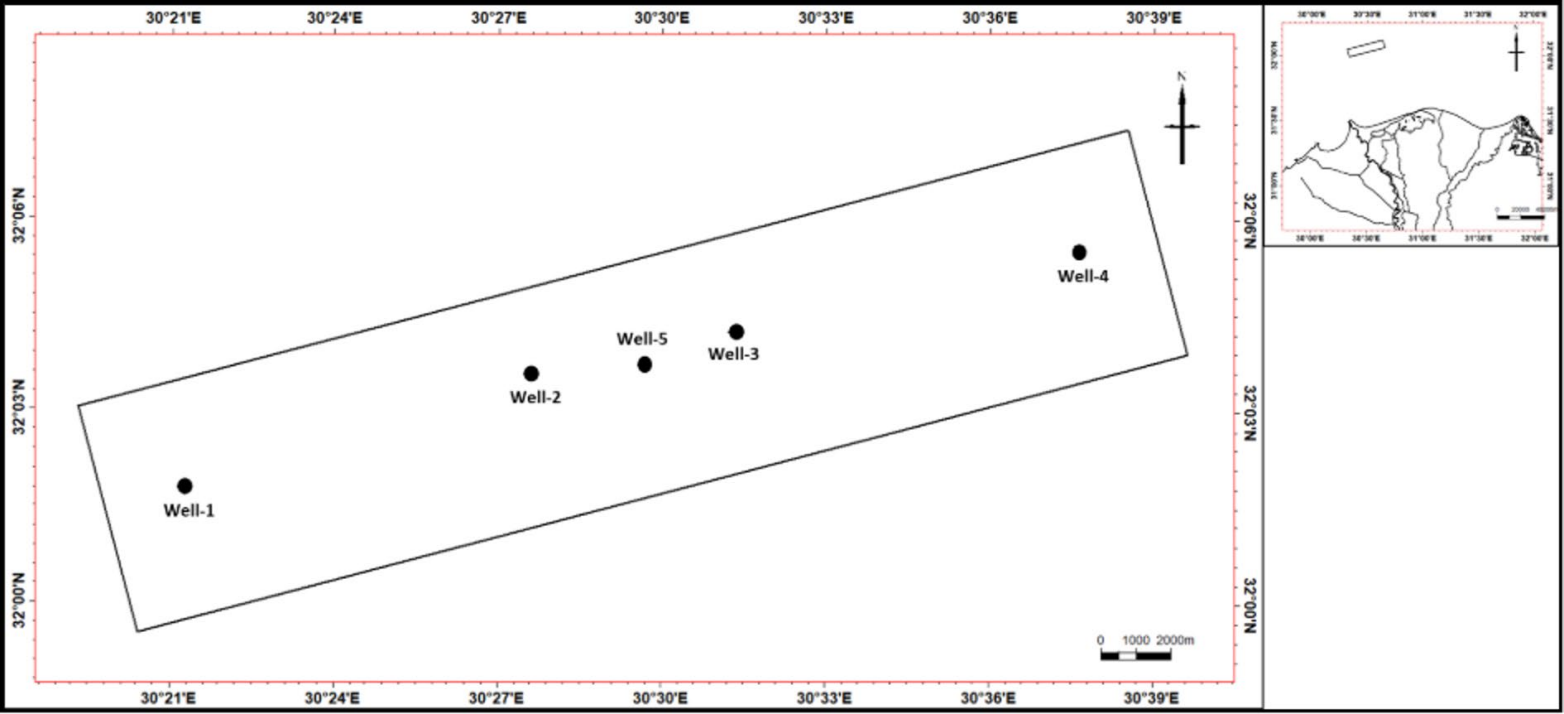
The location of study area with the distribution of the wells.

The distribution of wells within the study area is shown in Fig. 4; wells 1, 2, 3, and 4 are input wells, while well 5 is the blinded well.

P-impedance, S-impedance, and density seismic simultaneous pre-stack inversion cubes were generated in the **first phase** in this work. We use this result to estimate the interval velocity, which is the initial input used for estimating pore pressure.

Angle stack seismic data was classified into three cubes: near stack (0–15 degrees), mid stack (15–30 degrees), and far stack (30–45 degrees). We need to evaluate the data to decide if it is suitable for the task, or if additional data conditioning is needed.

**Fig. 5:**
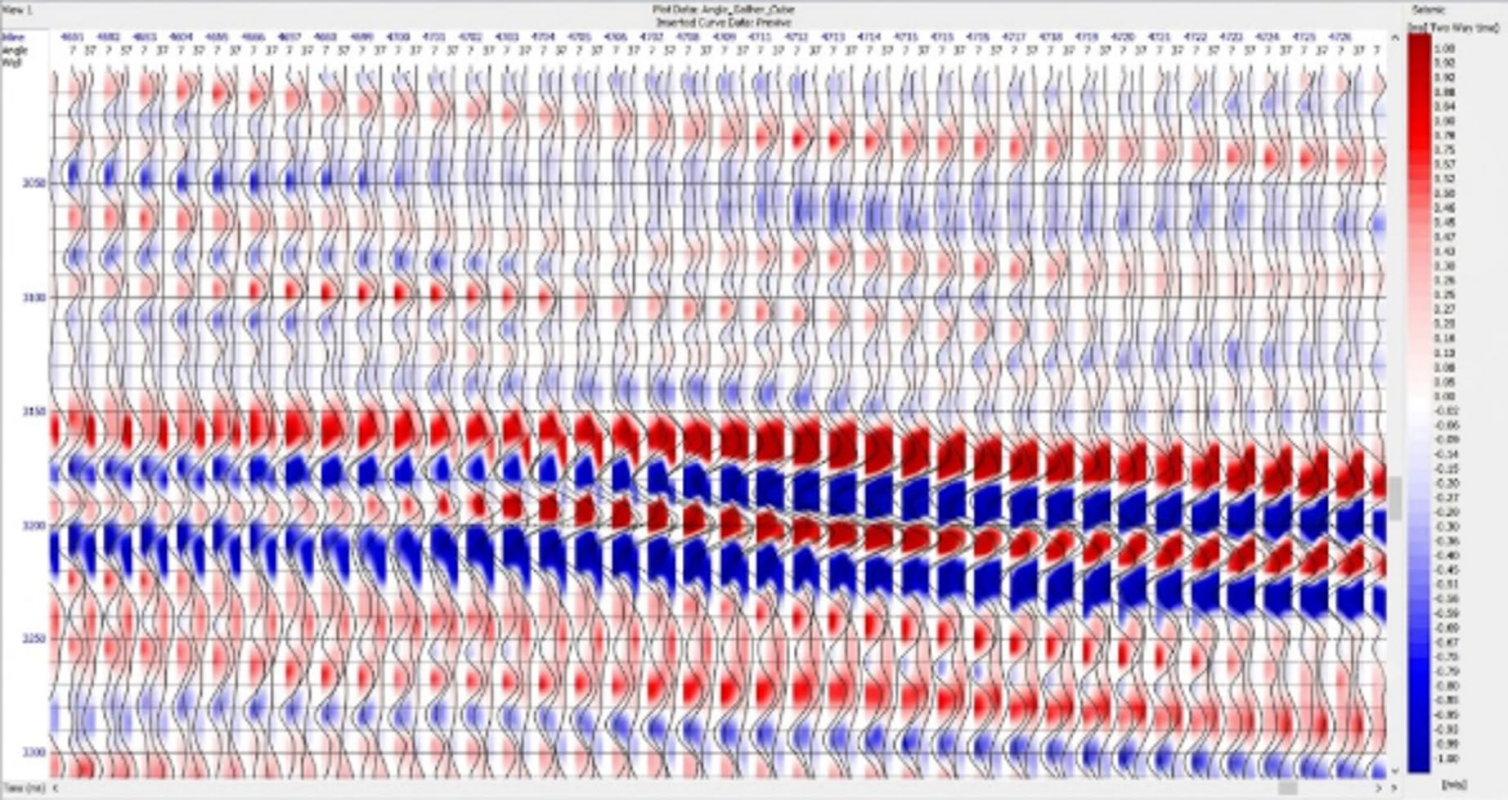
The gathered cube from the pre-stack seismic cubes at 7.5^o^, 22.5^o^ and 37.5^o^.

After the analysis of the angle stack gather (Fig. 5), we needed some data conditioning, including amplitude, frequency, and trim static balancing. Results of the data conditioning are displayed in the following figures.

**Fig. 6:**
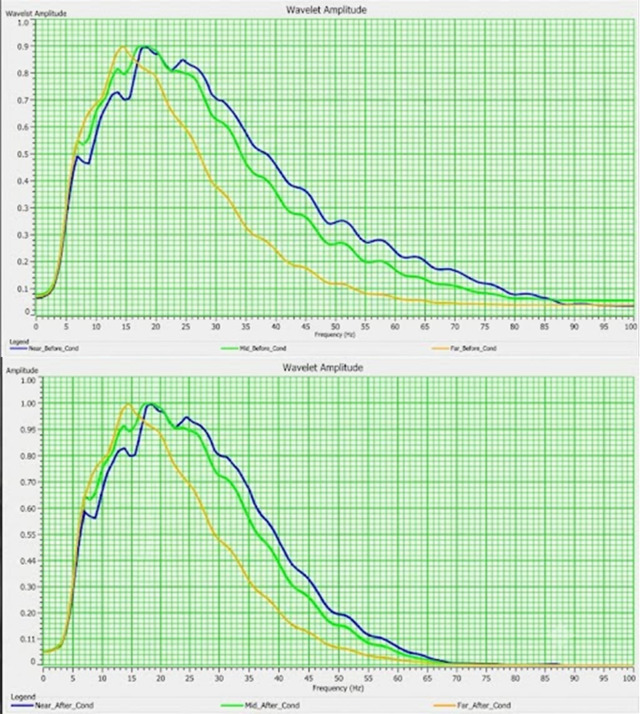
Amplitude spectrum for the pre-stack seismic data near (blue), mid (green) and far (orange) before (Up) and after (Down) the frequency filter. Extracted from 1400–3500 ms.

Frequency balancing is the first process in data conditioning to ensure that the three angle stacks have the same frequency range. The designed band-pass frequency filter has a high-frequency cut between 35 and 70 Hz and a low-frequency cut between 0 and 7 Hz. The following figure shows the angle stack frequency before and after the filter. Figure 6.

**Fig. 7:**
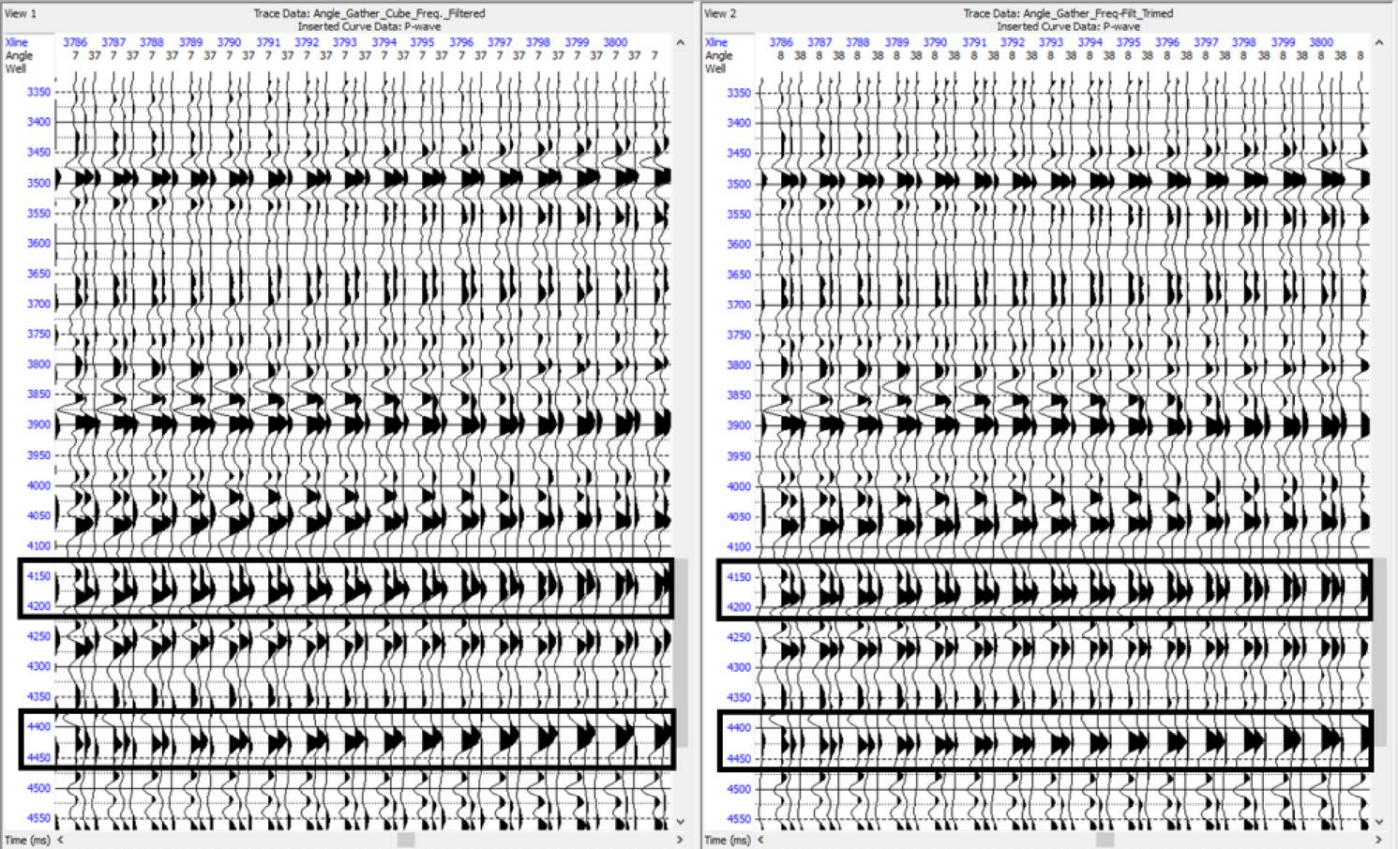
The comparison between the frequencies balanced gathered cube before and after trim static (right). The difference can be clear within the black rectangle.

Evaluating the time shift between the angles occurs next after the frequencies have been balanced. This effect is caused by changes in the angle of incidence over time, which causes a delay in a far angle relative to the near angles. If the issue occurs, time alignment is necessary for the data to correctly identify the event’s time. The following figure displays the trim static’s result. Figure 7.

**Fig. 8:**
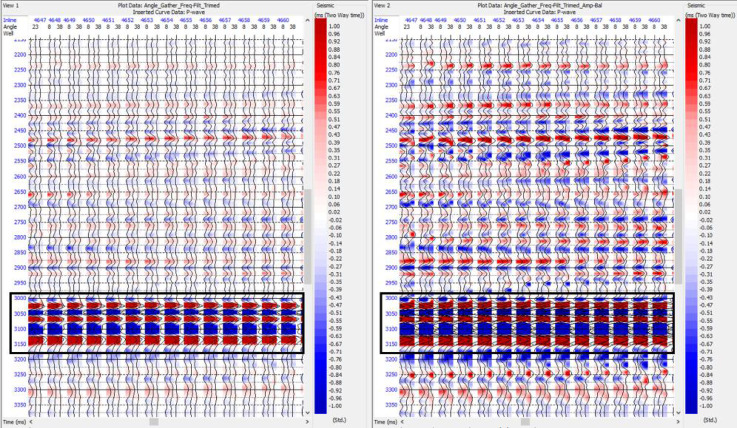
The comparison between the frequency-balanced- trimmed gathered cube before amplitude balancing (left) and frequency-balanced-trimmed gathered cube after amplitude balancing (right). The difference can be clear within the black rectangle.

The final step of the pre-stack seismic data conditioning technique is amplitude balancing. The objective of this step is to match the RMS amplitude of the theoretical response generated from the AVO synthetic data produced from the wells with the RMS amplitude of the real background interval of the seismic data. The angle stacks following amplitude balancing are shown in the following figure. Figure 8.

The wavelet extraction process for the pre-stack inversion was constrained by performing well-to-seismic ties across the near, mid, and far-angle data volumes. The following table presents the extraction parameters and the achieved correlation coefficients, demonstrating the quality of the well-seismic calibration. Table 1.

**Table 1:**
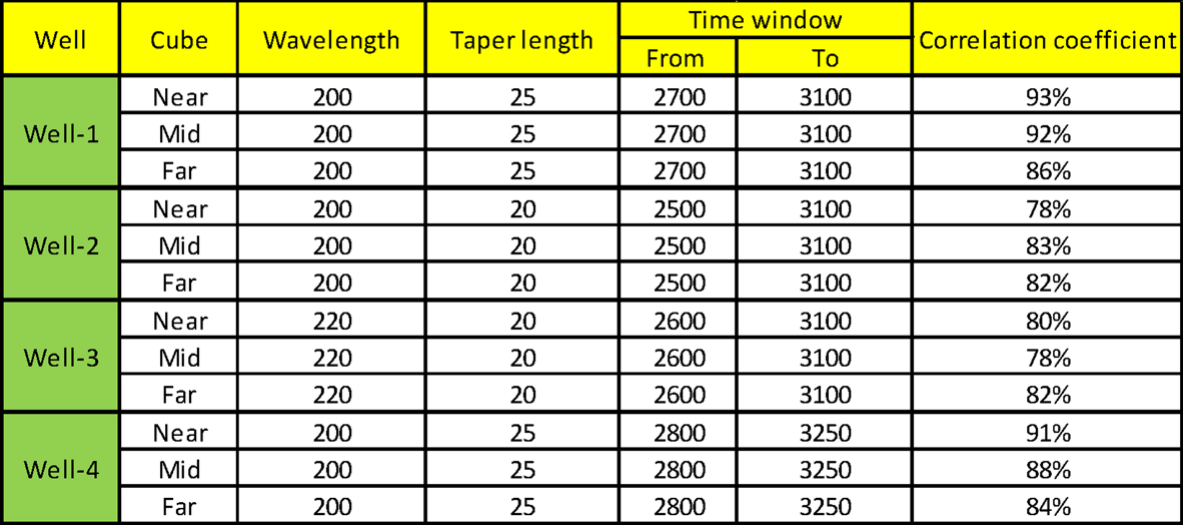
The extracted wavelet parameter and correlation coefficient for pre-stack inversion.

**Fig. 9:**
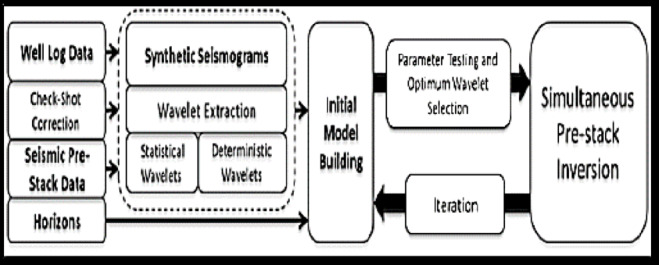
Flow chart outlining the steps of the simultaneous pre-stack inversion workflow (Ali, 2013).

We develop P-impedance, S-impedance, and density cubes from the angle stack using the approach displayed in the following figure. These cubes are then utilised to build the interval velocity, which is the first input for the pore pressure cube. Figure 9.

**Fig. 10:**
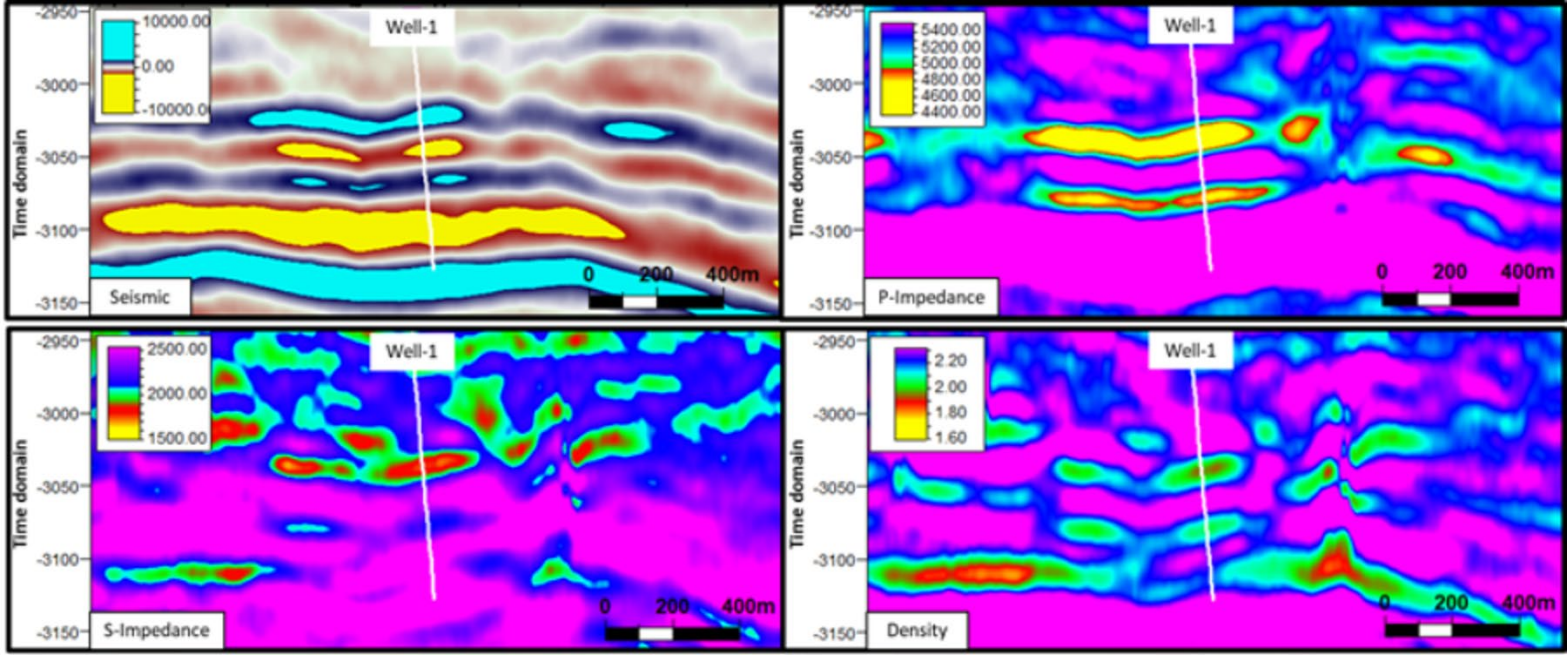
Pre-stack inversion results shown in arbitrary line on well-1; (1) seismic section, (2) P-impedance, (3) S-impedance, and (4) density.

To ensure the quality control (QC) of the simultaneous pre-stack inversion prior to interval velocity extraction, the following figure displays the inversion results for Well-1. Figure 10.

**Fig. 11:**
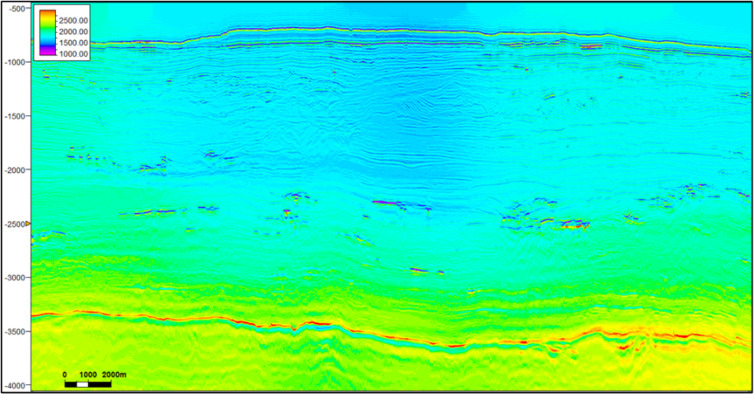
The calculated interval velocity from the P-impedance of the pre-stack inversion in the time domain.

The interval velocity produced by the simultaneous pre-stack inversion process is displayed in the following figure. Figure 11.

**Fig. 12:**
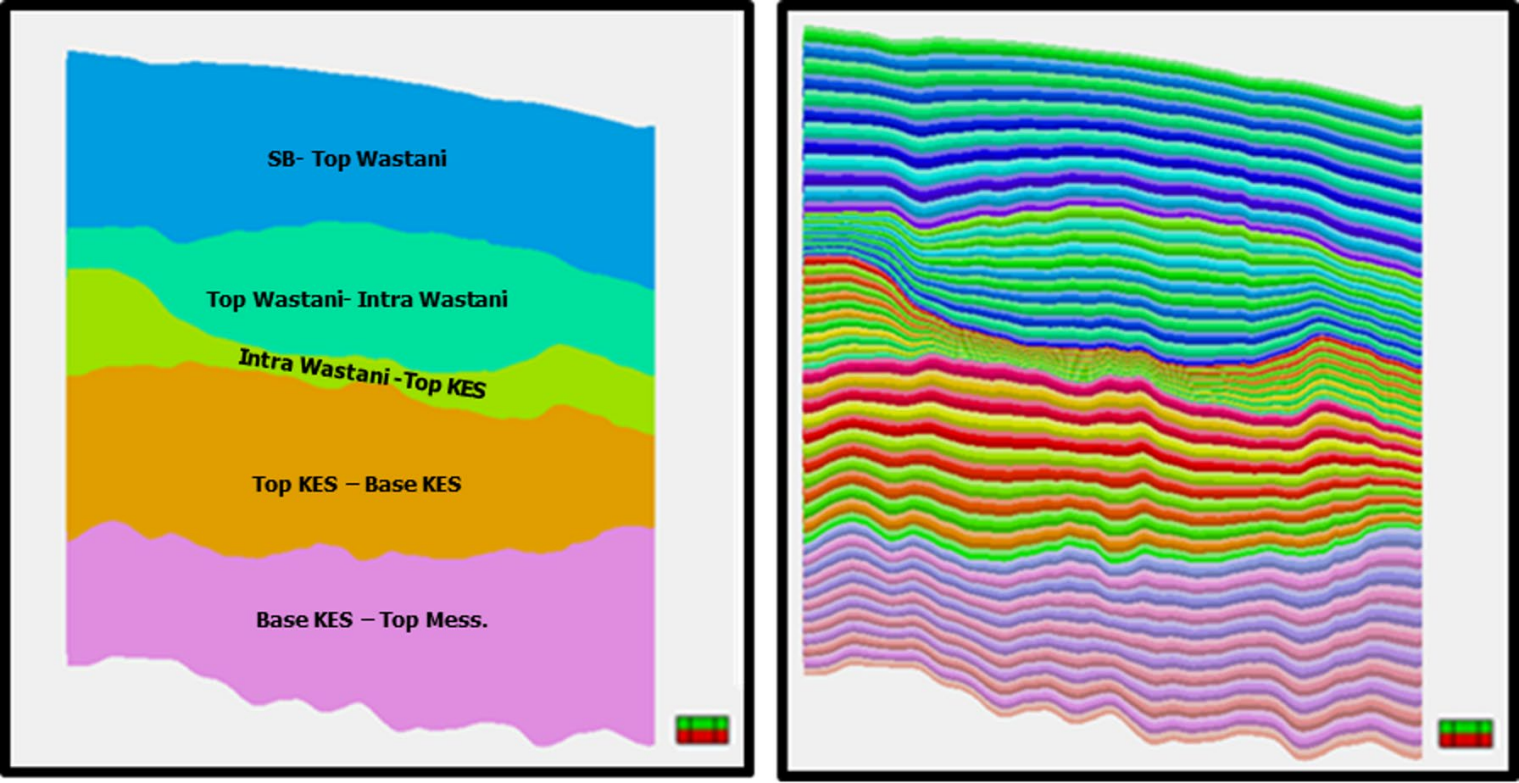
Model Zones in the left and the model layers in the right.

The second step involves using the workflow shown in Fig. 3 to estimate the pore pressure based on the produced interval velocity. To make calculations easier, the model was divided it into zones and layers, then the velocity values are then resampled into the model’s layers and cells. as shown in the following figures. Figure 12.

**Fig. 13:**
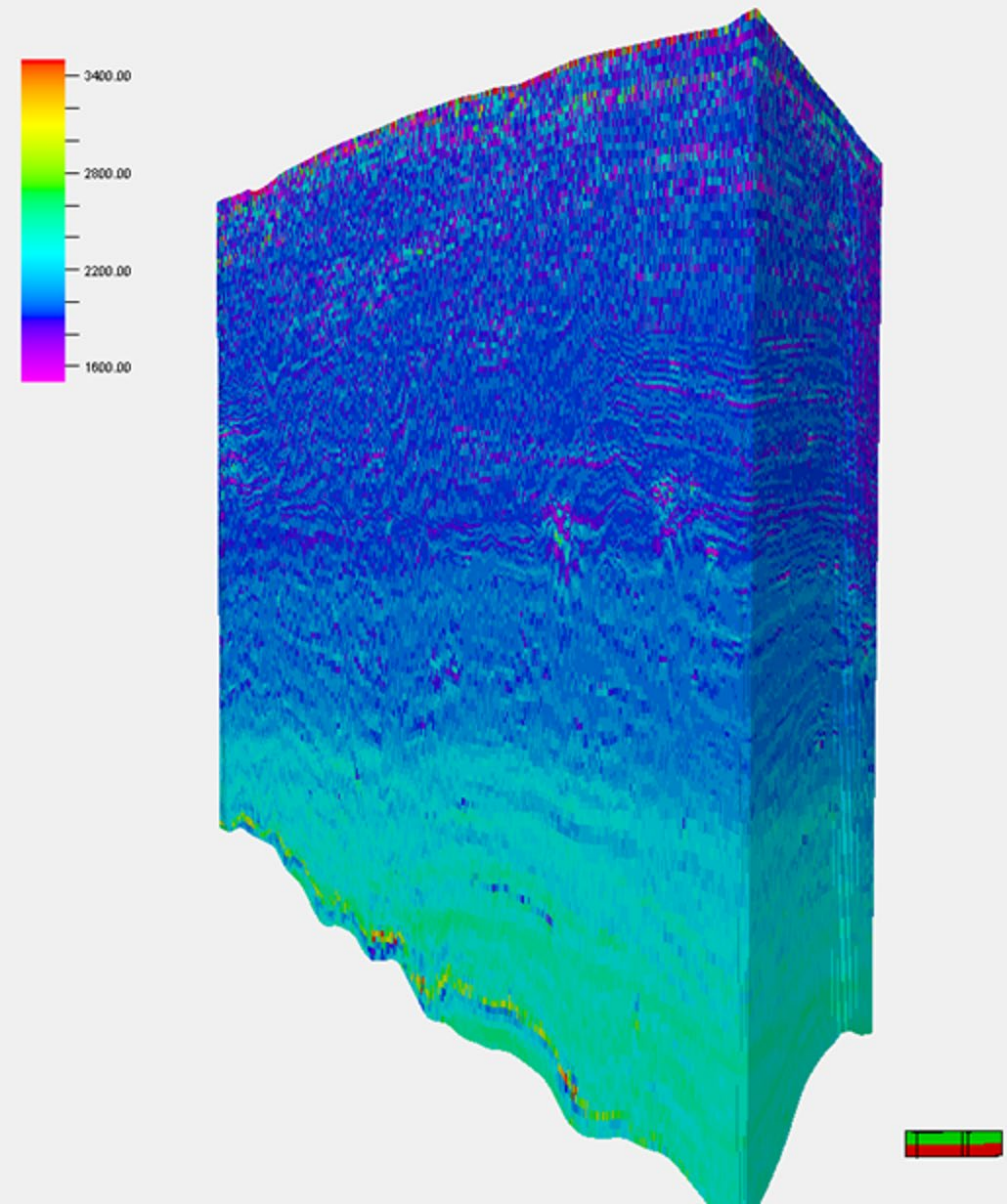
The interval velocity resampled at the model.

Building the Pore pressure workflow is the third phase. Predicting density in the shallow section for use in overburden prediction is the first step in the technique. Miller and Gardner’s equations are considered as the primary method used for density prediction. The Gulf of Mexico basin’s constant parameters are critical to the Miller and Gardner equations. In the study, we integrate the Miler and Gardner techniques to adjust the parameter to match the Nile delta and estimate the density log using excel sheet to predict the pressure logs and optimize the parameter which used in pressure cube prediction. The Gardner equation’s optimised parameter is displayed in the following table. Table 2.

**Table 2:**
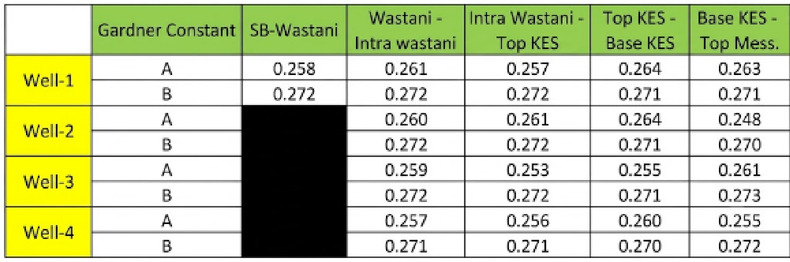
The optimum parameters of A and B within the different.

The following figure displays the predicted density cube. It uses the miller method up to 600 m BML and the Gardner technique for the rest of wells.

**Fig. 14:**
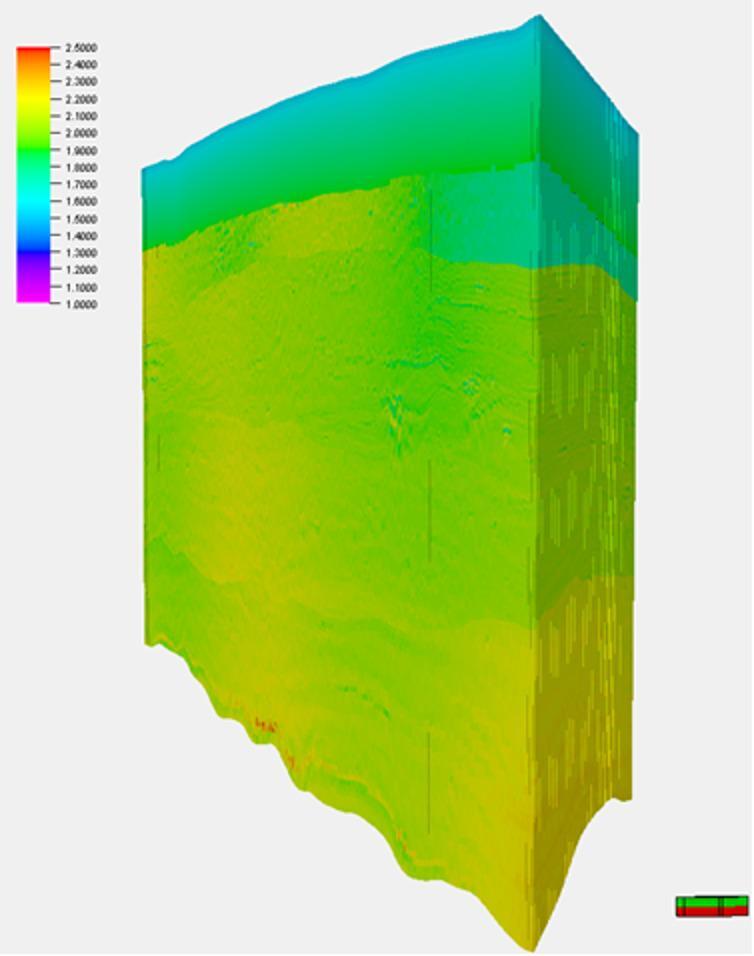
Final density cube is generated by combining the Miller and Gardner density cubes.

The final density cube produced by the Miller and Gardner methods is used in the OBP calculation (Fig. 13). As observed in Fig. 14, we can generate the final OBP cube by applying an automated process by combining the water header and sediment.

**Fig. 15:**
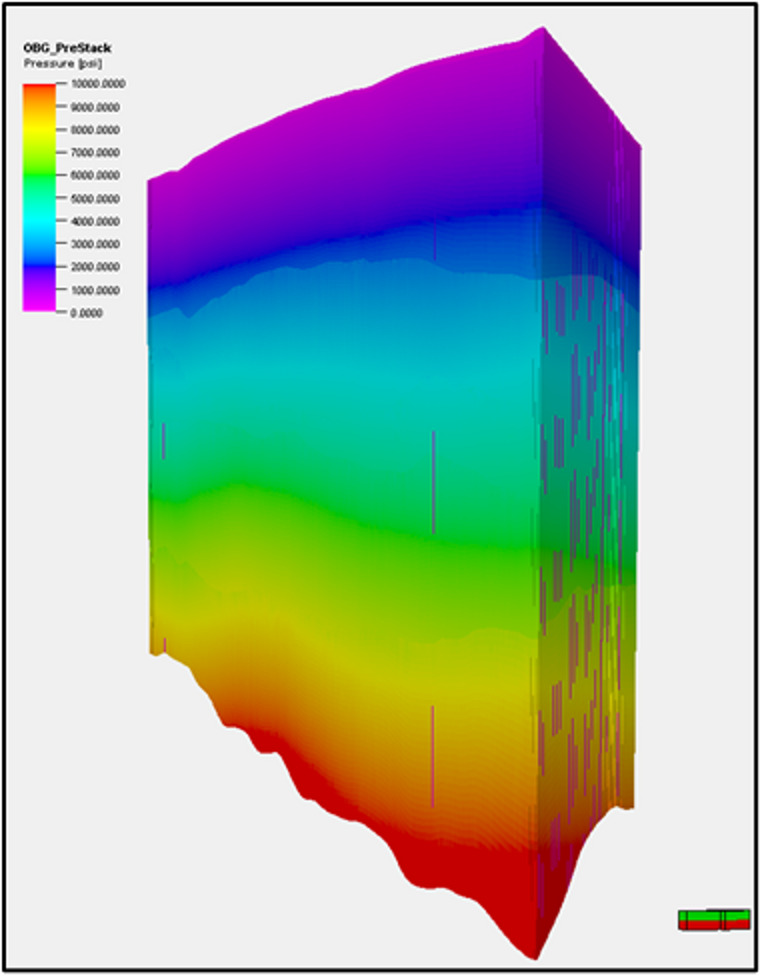
Final overburden pressure cube in psi.

As part of our process, we will now compare the actual data recorded at the rig site with the predicted data to verify its accuracy. In order to confirm the accuracy of our prediction, we checked both the input wells and the blind well (well-5) during this step. The OBP cube output and the actual data recorded at the rig site are presented in Fig. 15.

**Fig. 16:**
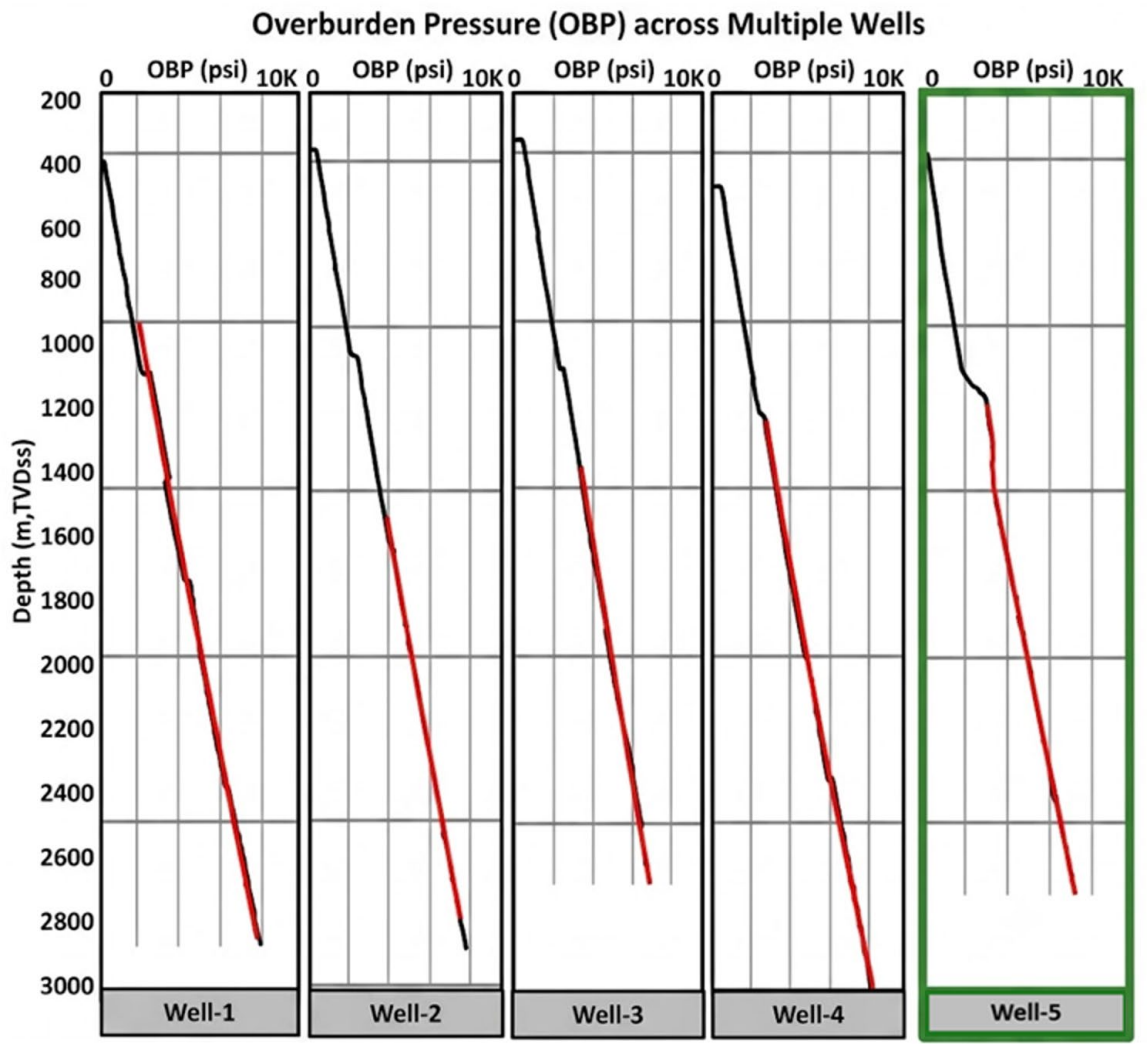
The comparison between the generated OBP (black) and the recorded data from the rig site (red), Well-5 represents the blind well (green frame).

The effective vertical stress is the second term in the Terzaghi equation. It is computed using the Bowers equation, which optimises the X value to match the Nile delta. The optimised X parameter is displayed in the following table. Table 3.

**Fig. 17:**
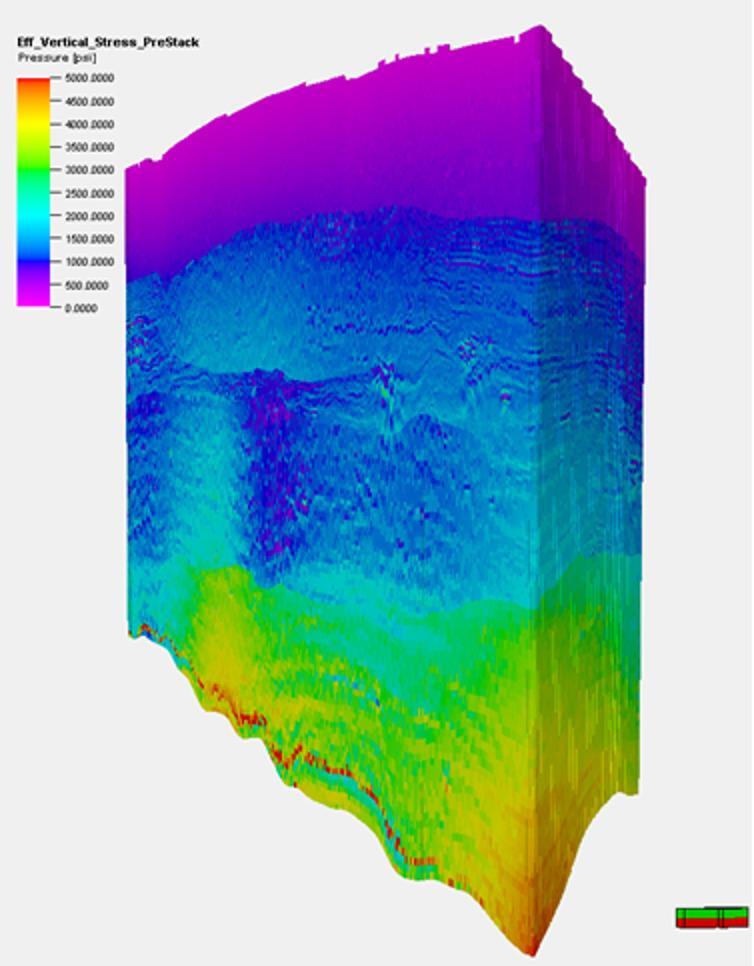
The final effective stress cube in psi.

Figure 16 displays the vertical stress cube’s final output, and Fig. 17 compares the VES cube result with the actual data recorded at the rig site (Actual OBP – Actual PP).

**Table 3:**
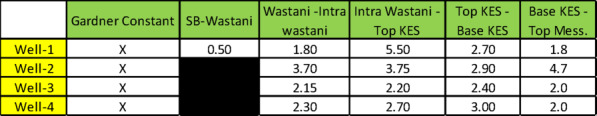
The optimum parameters X within the different zones.

**Fig. 18:**
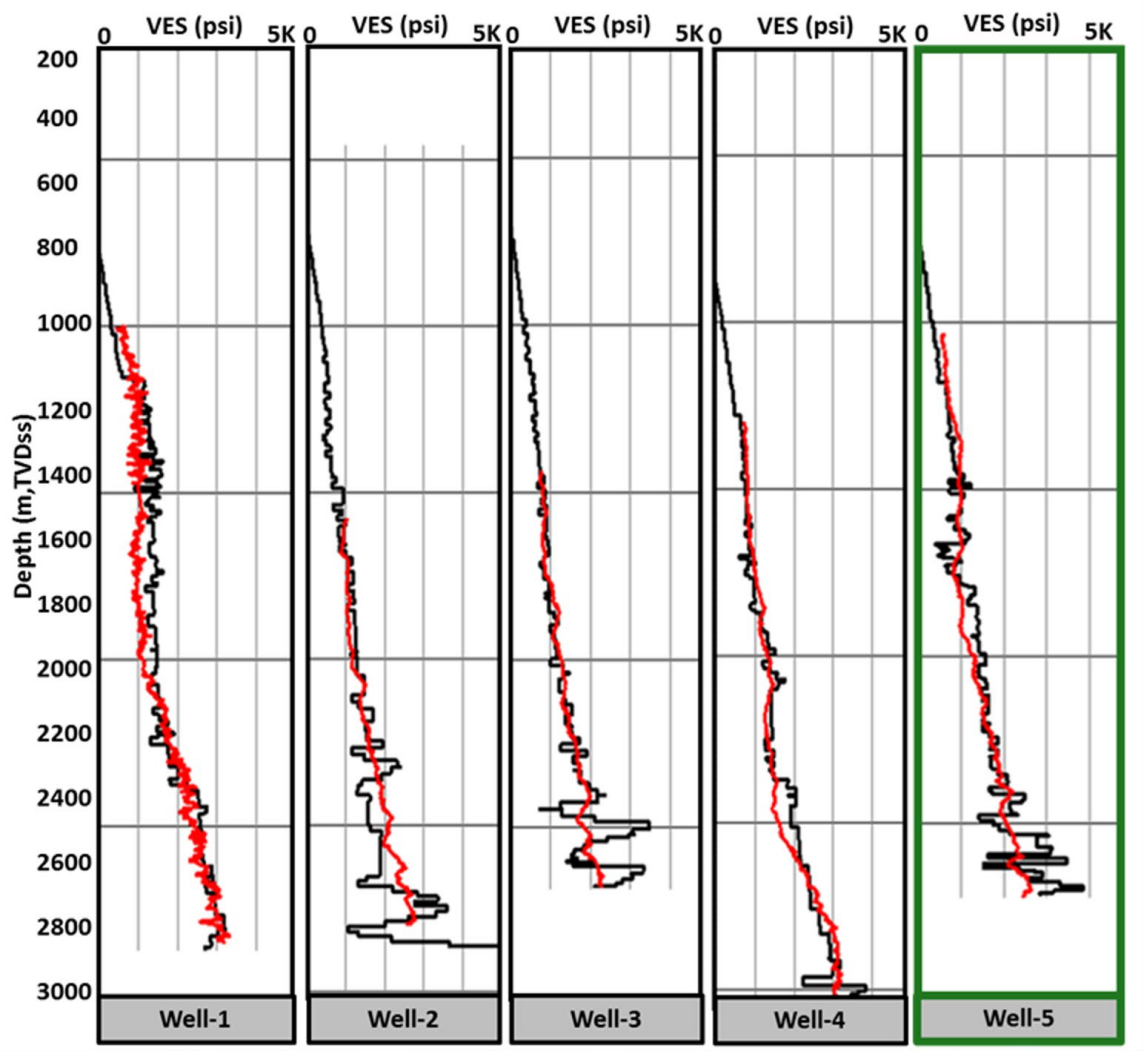
The comparison between the generated σv (black) and the difference between actual OBP and actual PP (red), Well-5 represents the blind well (green frame).

**Fig. 19:**
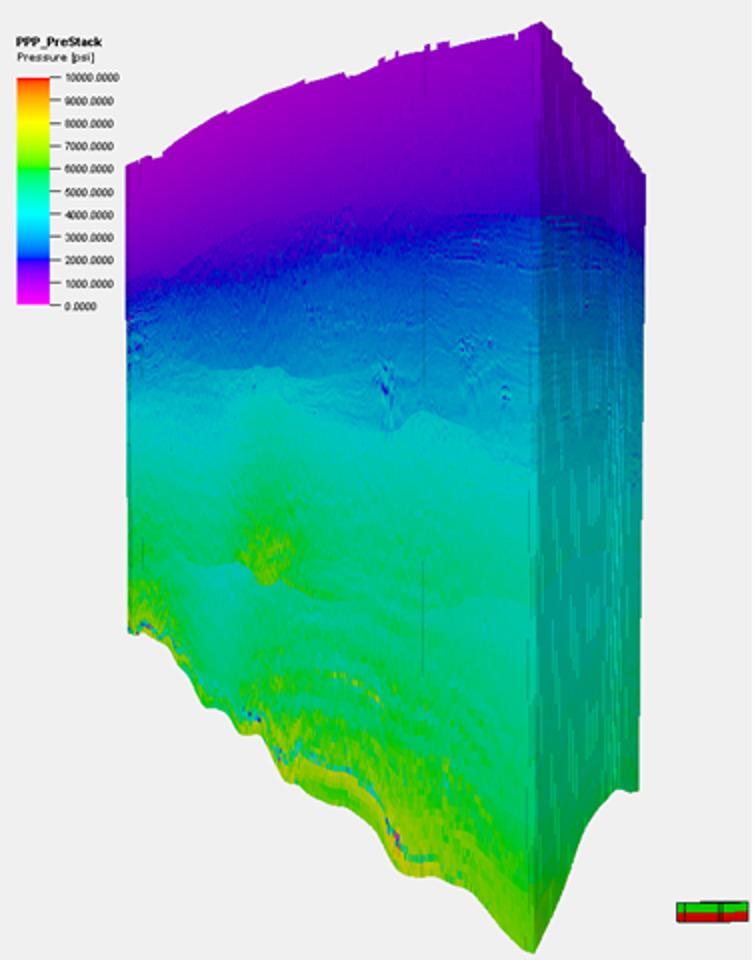
The final pore pressure cube in psi.

**Fig. 20:**
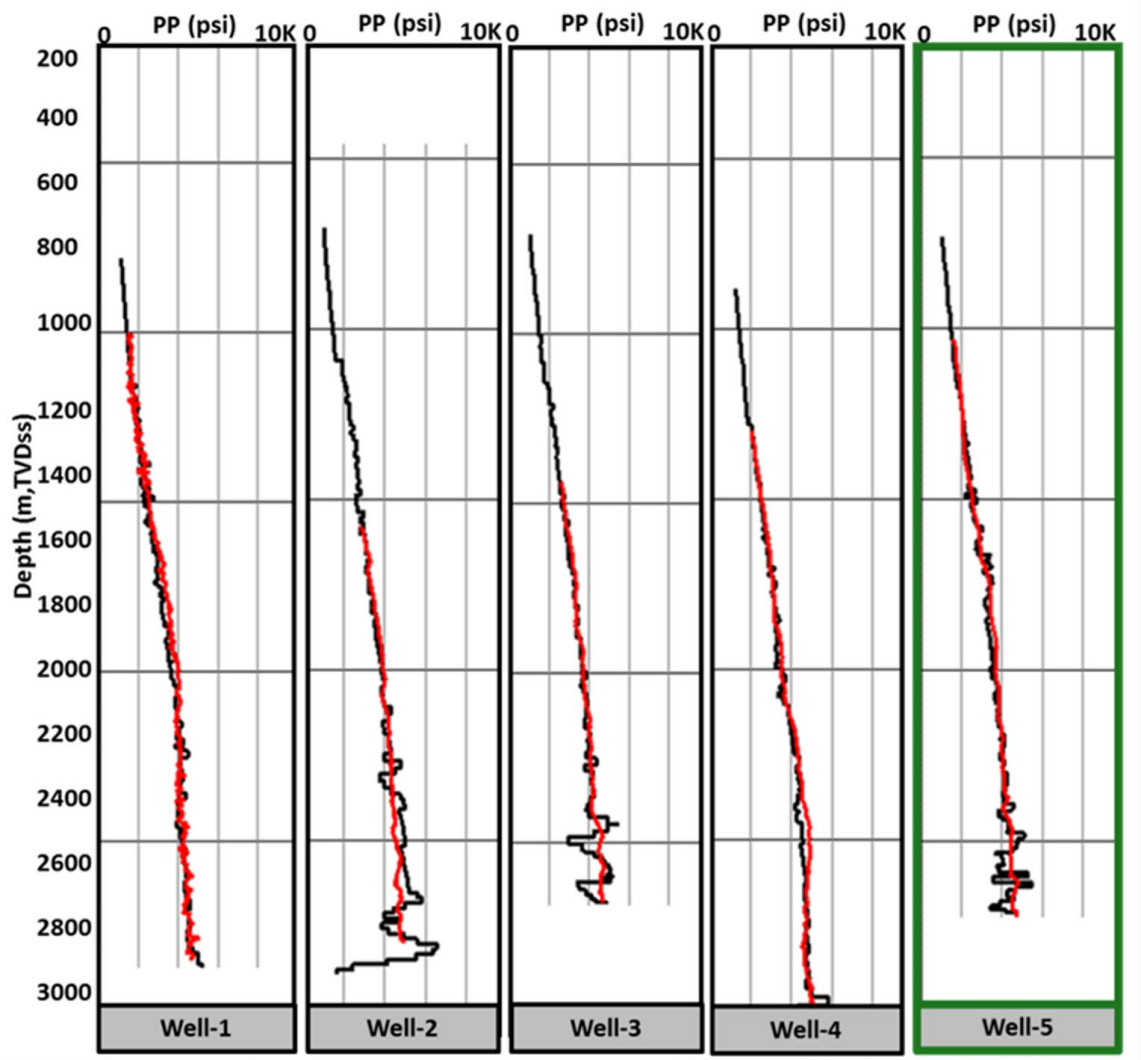
The comparison between the generated PP from cube (black) and the actual PP from rig site (red), Well-5 represents the blind well (green frame).

**Fig. 21:**
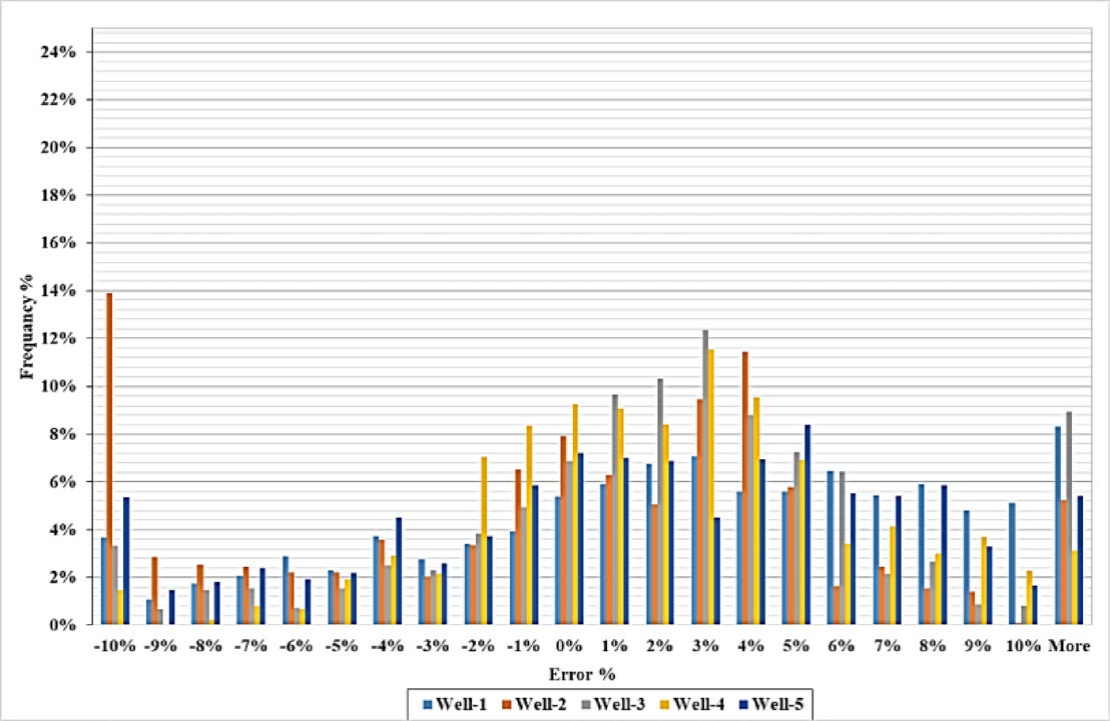
The error percentage of the pore pressure prediction from the pre-stack inversion interval velocity at the five wells.

We may get the pore pressure in 3D using the Terzaghi equation after calculating the OBP and VES. The pore pressure cube and a comparison between the actual PP data gained at the rig site and the PP cube result are displayed in the following figures. Figures 18, 19, 20, 21. 

As we displayed the result in qualitative, now we will show the result in quantitative. The error percentage calculated between the actual pore pressure recorded at the rig site using LWD data (GR & resistivity) and the estimated pore pressure generated from interval velocity derived from pre-stack inversion.

The error at the wells is estimated using a method that includes producing a histogram to evaluate the frequency and common range of errors in the data. The error is calculated by subtracting the actual pore pressure from the predicted pore pressure.

The following figures display the PP error calculations for the PP product generated from the cubes for all well.

## Summary & conclusion

The work presented in this paper focuses on the 3D pressure cube prediction technique, which can be used for future wells without needing the development of a workflow for each well at future campaigns. By predicting the overpressure zones, it may be helpful in an analysis of pressure behaviour during various geological ages.

The four wells are used as inputs in the paper’s work to build the model and optimise the constant for the Eaton and Bowers equations. To verify the model’s accuracy, we used the Blinded well, which was dropped out of the model-building process.

The workflow’s ability to predict the pressure for future wells in the same field is confirmed by the predicted pressure cubes. Additionally, it may be used to accurately generate pressure cubes in the offshore Nile delta or any other comparable basin.

The pre-stack inversion process includes different steps, including data gathering, well-to-seismic tie, seismic data conditioning, initial model building, and the final pre-stack inversion model. The standard process for pre-stack inversion is to evaluate reservoir characteristics using wireline logs. However, in our study, the inversion was done on the shale interval, which is not completely covered by the wireline logs. In addition, the main challenge with this process is the target intervals. We aim to build the pre-stack inversion cube at the shale intervals compared to the normal workflow, which generates at the sand interval.

The result of the workflow is the pore pressure, which is generated in the shale intervals by the pre-stack inversion process. The pore pressure prediction’s final result could show slightly greater errors mainly due to the multi-step process that includes pre-stack inversion. If there is a minor error at the start of the workflow, it will gradually accumulate until we reach the final stage. This method generates an accurate estimation of pore pressure with an acceptable range of error.

## Data Availability

The data that support the findings of this study are available from Rashid Petroleum company but restrictions apply to the availability of these data, which were used under license for the current study, and so are not publicly available. Data are however available from the authors upon reasonable request and with permission of Egyptian General Petroleum Company (EGPC).
